# The bread and butter of statistical analysis “t-test”: Uses and misuses

**DOI:** 10.12669/pjms.316.8984

**Published:** 2015

**Authors:** Younis Skaik

Statistical tests are very important in biomedical research.^1^ Several factors play a role in selecting the most appropriate statistical test.^2^ The misuse or inaccurate use of a statistical test may navigate the research in the wrong direction, and hence incorrect conclusions. Because it is probably the most commonly used statistical test, Student’s t-test is considered “the bread and butter” of statistical analysis. The William Gossett test “Student’s t-test” is easy to use, however, it is also misused.^3^ There are three types of the t-test, which are used for comparing either a single mean or two population means (Table-I). Each t-test can be used under specific conditions and criteria.

**Table-I:** Types of Student’s t-test.

Type of t-test	Test Description
One-sample t-test	To compare a single mean to a fixed number or gold standard
Two-Sample t-test	To compare two populations means based on independent samples from the two populations or groups
Paired t-test	To compare two means based on samples that are paired in some way

## Types of t-test

## 1. One- Sample t-Test

It is used for comparing sample results with a known and specified value, sometimes a “gold standard”. The task of this test should be to answer the question “is the mean of the population from which the sample is taken is different from the specified value”? For example, based on a random sample of 200 students, can we conclude that the average IQ score this year is lower than the average from 3 years ago?

In most studies, a sample size of at least 40 can guarantee that the sample mean is approximately normally distributed, and the one-sample t-test can then be safely applied.

## 2. Two-Sample t-test

It is used to know whether the unknown means of two populations are different from each other based on independent samples from each population. To apply this test, it is very important that the two samples are independent and unrelated to each other. The samples can be obtained from two separate populations, or from a single population that has been randomly divided into two groups, and each group subjected to one of two treatments. The test is only valid for comparing means from a quantitative variable.

## 3. Paired t-test

It is appropriate for data in which the two samples are paired in some way, such as the following examples.

**3-1** Pairs consist of before and after measurements on a single group of subjects.

**3-2** Two measurements on the same subject (e.g., right and left arm) are paired.

**3-3** Subjects in one group (e.g., those receiving a treatment) are paired or matched on a one-to-one basis with subjects in a second group (e.g., control subjects).

## Misuses of t-tests: Please do not use t-tests in the following cases.

1. If the sample size is small (less than 15), the one-sample t-test should not be used if the data are clearly skewed or the outliers are present. Nonparametric test can be performed.
2. If the sample size is moderate (at least 15), the one-sample t-test should not be used if there are severe outliers.
3. If the outcome measure is categorical (nominal/discrete) variable such as, gender, and even if the data have been numerically coded, the two-sample t-test should not be applied.
4. If a group of subjects receives one treatment, and then the same subjects later receive another treatment. This is a paired t-test and not two-sample t-test.
5. If subjects receive a treatment, and then the results are compared to a known value (often a “gold standard”). This is a one-sample t-test and not two-sample t-test.
6. If the study aims to compare three or more means, then it is better to use an analysis of variance to avoid the loss of control over the experiment-wise significant level.
